# Enzymatic post-treatment of ozonation: laccase-mediated removal of the by-products of acetaminophen ozonation

**DOI:** 10.1007/s11356-023-25913-w

**Published:** 2023-02-28

**Authors:** Dorothee Schmiemann, Lisa Hohenschon, Indra Bartels, Andrea Hermsen, Felix Bachmann, Arno Cordes, Martin Jäger, Jochen Stefan Gutmann, Kerstin Hoffmann-Jacobsen

**Affiliations:** 1 Department of Chemistry and Institute for Coatings and Surface Chemistry, Niederrhein University of Applied Sciences, Adlerstr. 32, 47798 Krefeld, Germany; 2 Institute of Physical Chemistry and CENIDE (Center for Nanointegration), University Duisburg-Essen, Universitätsstraße 5, 45141 Essen, Germany; 3 Present Address: Wfk-Cleaning Technology-Institute e.V., Campus Fichtenhain 11, 47807 Krefeld, Germany; 4 Faculty of Chemistry, Instrumental Analytical Chemistry, University of Duisburg-Essen, Universitätsstraße 5, 45141 Essen, Germany; 5 Present Address: Institute of Theoretical Chemistry, University Duisburg-Essen, Universitätsstraße 5, 45141 Essen, Germany; 6 ASA Spezialenzyme GmbH, Am Exer 19C, 38302 Wolfenbüttel, Germany; 7 Deutsches Textilforschungszentrum Nord-West gGmbH, Adlerstr. 1, 47798 Krefeld, Germany

**Keywords:** Wastewater post-treatment, Micropollutants, Organic trace contaminants, Acetaminophen, Ozonation, Laccase, Toxicity, Mass spectrometry, Quinone, Polymerization

## Abstract

Ozonation is a powerful technique to remove micropollutants from wastewater. As chemical oxidation of wastewater comes with the formation of varying, possibly persistent and toxic by-products, post-treatment of the ozonated effluent is routinely suggested. This study explored an enzymatic treatment of ozonation products using the laccase from *Trametes versicolor*. A high-performance liquid chromatography coupled with high-resolution mass spectrometry (HPLC-HRMS) analysis revealed that the major by-products were effectively degraded by the enzymatic post-treatment. The enzymatic removal of the by-products reduced the ecotoxicity of the ozonation effluent, as monitored by the inhibition of *Aliivibrio fischeri.* The ecotoxicity was more effectively reduced by enzymatic post-oxidation at pH 7 than at the activity maximum of the laccase at pH 5. A mechanistic HPLC-HRMS and UV/Vis spectroscopic analysis revealed that acidic conditions favored rapid conversion of the phenolic by-products to dead-end products in the absence of nucleophiles. In contrast, the polymerization to harmless insoluble polymers was favored at neutral conditions. Hence, coupling ozonation with laccase-catalyzed post-oxidation at neutral conditions, which are present in wastewater effluents, is suggested as a new resource-efficient method to remove persistent micropollutants while excluding the emission of potentially harmful by-products.

**Graphical abstract:**

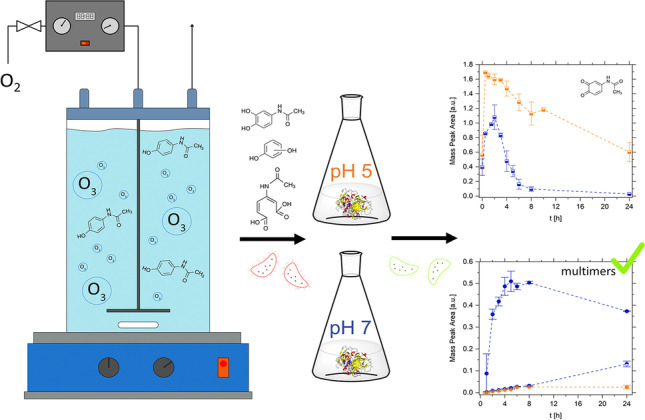

**Supplementary information:**

The online version contains supplementary material available at 10.1007/s11356-023-25913-w.

## Introduction

The presence and persistence of organic trace contaminants in the aquatic environment have raised increasing concerns in the past two decades. Among these micropollutants, pharmaceuticals were detected worldwide in the effluents of wastewater treatment plants in a concentration range from nanograms to micrograms per liter (Fatta et al. 2007; Lacey et al. 2008; Heberer 2002). Conventional wastewater treatment systems do not efficiently remove micropollutants, so these persistent substances accumulate in the aquatic environment (Monteiro and Boxall 2010; Zenker et al. 2014). Prompted by the increasing amount of evidence on adverse effects on the health of aquatic organisms (e.g., feminization of fish) and on the quality of drinking water, tertiary treatment for the abatement of the micropollutant load from sewage plant effluents is currently discussed in many industrialized countries (Alder et al. 2006; Huerta et al. 2012).

Various techniques have been studied from the laboratory to the full scale (Voigt et al. 2020) including extended biological treatment processes (Abejón et al. 2015, Ahmed et al. 2017, Cecconet et al. 2017), adsorption (Tijani et al. 2013; Sharif et al. 2013; Gao et al. 2012; Yu et al. 2016), chemical oxidation, and advanced oxidation processes (AOPs) (Gogoi et al. 2018; Oppenländer 2003; Andreozzi 1999; Voigt et al. 2018). Pilot studies have proven that ozonation is one of the most effective techniques for micropollutant elimination on a technical scale (Lee et al. 2013). However, the mineralization of organic compounds does generally not occur. This can lead to potentially toxic transformation products (Fatta-Kassinos et al. 2011; Andreozzi et al. 2003; Völker et al. 2019). Therefore, post-treatment is recommended, usually by (biological) filtration. However, biological filtration suffers from the low nutritive value of the treated wastewater.

Enzymatic transformation of recalcitrant organic contaminants is a promising eco-friendly concept (Durán and Esposito 2000; Barber et al. 2020; Alshabib and Onaizi 2019; Haugland et al. 2019; Stadlmair et al. 2018). Among these enzymes, laccases are of particular interest as these enzymes only require molecular oxygen as a co-substrate and can oxidize a wide range of pollutants (Ikehata et al. 2004; Arregui et al. 2019; Gasser et al. 2014) which can be extended by the use of mediators (Baiocco et al. 2003; Morozova et al. 2007). The organic compounds are oxidized at the *T*_1_ Cu center to radicals (Piontek et al. 2002), which further react via polymerization by oxidative coupling, chemical rearrangement, or, depending on the relative redox potentials of the reaction mixture, redox shuttle (Jeon et al. 2012; Sun et al. 2013; Cañas and Camarero 2010).

Laccase treatment has been coupled with membrane technology (Lloret et al. 2013; Hahn et al. 2018), persulfate oxidation (Asif et al. 2020), and adsorption (Nguyen et al. 2016).

This study explores the combination of ozonation with enzymatic post-treatment for complete remediation of wastewater containing trace contaminants. Acetaminophen (APAP) is used as a model compound. It is the most widely used pain medicine in the world and hence detected in surface water, groundwater (Kaufman et al. 2002), and sewage plant effluent (Alygizakis et al. 2016; Phong et al. 2016) samples all over the world. APAP ozonation has been studied before (Skoumal et al. 2006; Villota et al. 2019; Hamdi El Najjar et al. 2014; Torun et al. 2015) and the parent drug itself is susceptible to laccase treatment (Skoumal et al. 2006; Villota et al. 2019; Ba et al. 2014; Lu et al. 2009).

APAP is ozonated in the first step and laccase from *Trametes versicolor* is used in the second, post-treatment step, to remove potentially harmful by-products of ozonation and residual APAP. The removal efficiency as well as the mechanism of enzymatic conversion of ozonation products are investigated by high-performance liquid-chromatography coupled with high-resolution mass spectrometry (HPLC-HRMS) analysis. Specifically, the effect of acidic and neutral pH on the mechanism and the process efficiency is investigated. Supporting kinetic and ecotoxicological analysis are used to develop recommendations for a safe chemo-enzymatic trace contaminant treatment.

## Materials and methods

### Materials

4-Acetaminophenol (APAP; 98%) and 1,4-benzoquinone (99%) were purchased from Acros organics (Geel, Belgium). 3-Hydroxy-acetaminophen (TP 168; 98%) was acquired from Hölzel (Cologne, Germany). 4-Hydroxy-3,5-dimethoxybenzaldehyde azine (syringaldazine (SGZ) ≥ 98%) was purchased from Sigma-Aldrich (Steinheim, Germany). Ammonium acetate (≥ 97%, p.a., ACS), acetic acid (100%; p.a.), methanol (HPLC Ultra Gradient Grade), acetonitrile (≥ 99.9%, LC–MS Grade), D( +)glucose monohydrate (≥ 99.5%, Ph. Eur.), sodium chloride (NaCl ≥ 99.5%, p.a., ACS), magnesium chloride hexahydrate (≥ 99%, p.a., ACS), potassium chloride (≥ 99.5%, p.a., ACS), and N-2-hydroxyethylpiperazine-N′-2-ethanesulfonic acid (HEPES ≥ 99.5%, p.a.) were obtained from Carl Roth (Karlsruhe, Germany). 3,5-Dichlorophenol (98%) was purchased from Alfa Aesar (Kandel, Germany). Ammonia solution (32%) was from Bernd Kraft (Duisburg, Germany). Formic acid (≥ 97.5%, LC–MS grade) was purchased from Fluka-Honeywell (Seelze, Germany). Milli-Q water was used to prepare all solutions (Simplicity 185, Merck Millipore, Billerica, MA, USA). The laccase from *Trametes versicolor* (*T. versicolor*) was produced by ASA Spezialenzyme GmbH (Wolfenbüttel, Germany) as described previously (Hahn et al. 2018). The company markets the enzyme under the name “laccase C.” *Aliivibrio fischeri* (*A. fischeri*) was obtained freeze-dried from LCK 488 (Hach Lange, Düsseldorf, Germany).

### Ozonation

Ozonation experiments were carried out in a 0.5-L batch reactor (DWK Life Sciences, Wertheim, Germany). Ozone was produced using a COM-AD-01/02 ozone generator (Anseros, Tübingen, Germany) with an oxygen flow of 25 L/h and a generator capacity of 2.8%. The ozone flow was continuously introduced through a glass frit into a batch reactor equipped with a magnetic stirrer. Ozonation of 50 mg/L APAP in 20 mM ammonium acetate buffer was performed for 30 min at pH 7 and room temperature (23 ± 2 °C). Ozonation kinetics were determined by multiple independent experiments. Each data point was determined at least in triplicate.

### Laccase assay

Laccase activity before and during the degradation experiment was measured by oxidation of syringaldazine. The assay was performed at pH 5 in ammonium acetate buffer with a concentration of 33 µM syringaldazine in the test mixture. The measurement was carried out with a UV-1650PC spectrophotometer (Shimadzu, Duisburg, Germany) at 530 nm and 20 °C.

Michaelis–Menten kinetics were monitored in a Spark plate reader (Tecan, Switzerland) with 96-well plates at 530 nm and 30 °C. In addition to the ammonium acetate buffer at pH 5 and pH 7, reference measurements were carried out with ozonated APAP solution and 3-hydroxyacetaminophen (TP 168) using a concentration of 55 µM and 27.75 µM, respectively.

### Treatment of ozonated APAP solution with laccase 

Laccase treatment was performed on an ozonated APAP solution at 50% degradation of the initial APAP concentration. Prior to laccase addition, residual ozone was removed by purging with oxygen. Nineteen-milliliter ozonated APAP solution was treated with 10 mg/L laccase solution (314 ± 9 U/L) in 100-mL Erlenmeyer flasks with rubber stoppers in a shaker at 100 rpm at 20 °C. Samples for chromatographic analysis were conserved by laccase inhibition via the addition of methanol (1:1 v/v). The degradation of TP 168 was performed accordingly. The kinetics were determined by multiple independent experiments. Each data point was determined at least in duplicate.

For the spectral absorption analysis, 10 mg/L pure reference compound, i.e., TP 168 and 1,4-benzoquinone, was treated with laccase as described above. Extracted samples were analyzed with a UV-1650PC spectrophotometer (Shimadzu, Duisburg, Germany) at the indicated reaction time.

### HPLC-HRMS analysis

Reversed-phase chromatographic analysis was performed using an Agilent 1200 Series HPLC system (Agilent Technologies, Inc., Waldbronn, Germany) equipped with an Eclipse Plus C18 (ZORBAX, 3.5 µm, 2.1 × 150 mm, Agilent Technologies, Inc., Waldbronn, Germany) as described previously (Voigt et al. 2021). Briefly, a gradient of the eluent system acetonitrile/water, both acidified with formic acid, was applied.

An Agilent 6530 Q-ToF mass spectrometer (Agilent Technologies, Santa Clara, USA) equipped with a Jet-Stream Electrospray Ion Source (ESI) was coupled to the HPLC system and used in the positive ion mode. The fragmentation voltage was set to 125 V. For MS/MS spectra, the collision energy was set to 30 eV. The HPLC–MS was controlled using Mass-Hunter Workstation B.06.00 (Agilent Technologies, Santa Clara, USA).

### Toxicity assessment

The acute toxicity test was based on DIN EN ISO 11348–1:2008 for the determination of the inhibitory effect of water samples on the light emission of *A. fischeri* (Deutsches Institut für Normung e.V. 2009). Initially, the samples were adjusted to pH 7 ± 0.3 with NaOH or HCl and a salinity of 20 g/L with NaCl solution. Prior to measurements, the samples and the *A. fischeri* were incubated at 16 °C and 100 rpm for 15 min. Subsequently, 0.5 mL of the bacterial suspension was pipetted into each of the 24-well plates, and the initial and the remaining luminescence after incubation with 0.5 mL sample at 16 °C were measured with a Spark plate reader (Tecan, Switzerland). NaCl and 3,5-dichlorophenol (9 mg/L) were used as references. Luminescence inhibition was analyzed according to DIN EN ISO 11348–1:2008 at 5, 15, and 30 min of incubation. Samples were diluted such that the measured inhibitions were in the quantitative inhibition range, i.e., 10–90%, but preferentially inhibitions from 20 to 80% were used for analysis (Deutsches Institut für Normung e.V. 2009). A sixfold dilution was used for the analysis of the ozonation and a 40-fold dilution for the analysis of the laccase treatment. Each sample was assayed at least in two wells.

### Statistical analysis

Variance homogeneity was examined using the two-sample *F*-test. The two-tailed *t*-test (variance homogeneity) and the two-tailed *t*-test according to Welch (variance inhomogeneity) were performed using the critical value approach and significance level of 0.05 (Supporting Information, 1.10).

## Results and discussion

### Ozonation of APAP

**Fig. 1:**
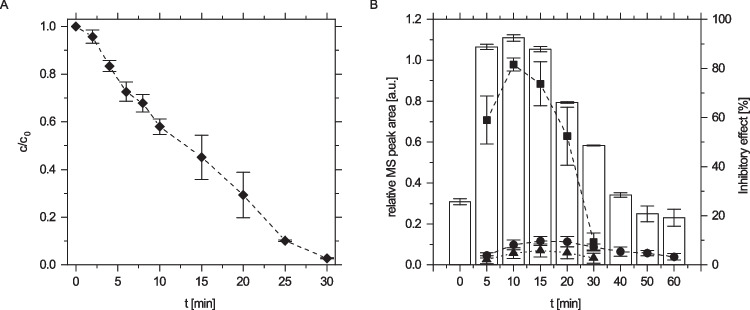
Kinetics of APAP ozonation: APAP degradation (**A**) and formation of transformation products and ecotoxicity as measured by the inhibitory effect on *A. fischeri* bioluminescence (**B**). The respective transformation products are indicated as squares (TP 168), triangles (TP 111), and circles (TP 200). Data points represent the mean normalized peak area of the replicate experiments ($\overline{{x }_{\mathrm{r}}}$). Error bars depict the standard deviation of the replicates (*s*_r_). Each data point was measured at least in triplicate. The inhibitory effect of the APAP solution during the degradation by ozonation on *A. fischeri* after 15-min contact time is shown as bars. Data points represent the mean value ($\overline{x }$) and error bars the standard deviation (SD) of the inhibition assayed in duplicate

Prior to treating the ozonation products of APAP with laccase from *T. versicolor*, the degradation as well as the formation of the transformation products (TPs) of APAP by ozonation were analyzed using HPLC-HRMS. Figure 1 shows the degradation of 50 mg/L APAP and the simultaneous formation of TPs. The low ozone flux leads to zero-order kinetics of APAP degradation. As determined by HRMS analysis and confirmed with previous studies, the suggested chemical structures of TPs are shown in Table 1. TP 111 and TP 168 were identified as phenolic compounds. TP 200 results from ring opening and leads to a potentially less harmful and readily degradable organic diacid. The ecotoxicity of the ozonation products was assessed via the inhibitory effect on the light emission of the fluorescent bacteria* A. fischeri*. As depicted in Fig. 1B, the ecotoxicity of the model wastewater increased after ozonation until 50% removal efficiency. The inhibitory effect correlated with the concentration of the phenolic transformation product TP 168 (Fig. 1B and Table S1). Although an ecotoxic effect of the 1,4-isomer of TP 111, hydroquinone, is also expected according to previous toxicological analysis (Qutob et al. 2022), TP 168 is the primary transformation product and, as a catechol, is expected to show lower biodegradability and higher sludge toxicity than TP 111. Hence, the degradation of TP 168 is of primary importance in order to produce an environmentally benign effluent.

**Table 1:**
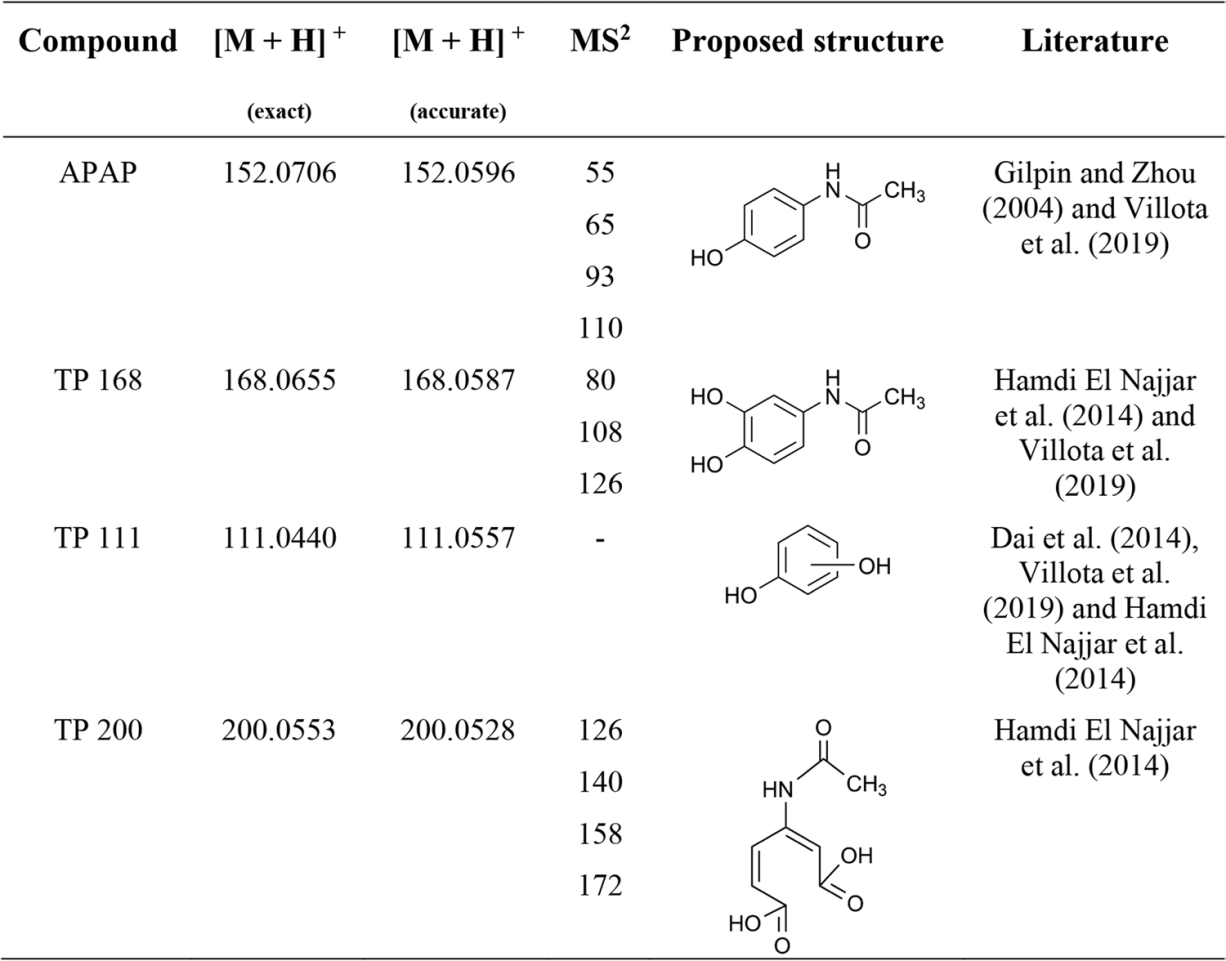
HRMS analysis of APAP and its degradation products (ESI +)

The exact and accurate masses of the [M + H]^+^ ion and its MS^2^ fragments after fragmentation are provided

### Degradation of APAP by laccase 

According to the activity maxima of fungal laccases (Margot et al. 2013), acidic conditions are typically applied for laccase treatment which would require the unfavorable addition of inorganic acids in a technical sewage treatment process. Also, a positive effect of neutral pH on APAP conversion has been discussed (Dai et al. 2014).

Therefore, we studied the enzymatic degradation of the ozonation transformation products under acidic (pH 5) and neutral (pH 7) conditions. A laccase-catalyzed post-treatment of ozonated APAP solution would include the elimination of residual APAP. Hence, the influence of pH on the kinetics of the degradation of APAP was analyzed in a preceding step. The degradation of APAP by laccase has been suggested to occur via the formation of radicals, which further react to polymers by oxidative coupling (Lu et al. 2009; Wu et al. 2020). Multimer formation is typically observed in a limited time frame at the beginning of the reaction as these intermediate products subsequently oligomerize to insoluble oligomers (Wang et al. 2018).

**Fig. 2:**
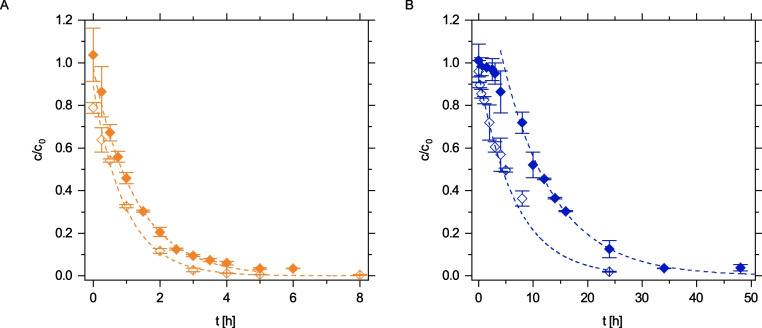
Degradation of APAP without (unfilled diamonds) and with previous ozone treatment (filled diamonds) at pH 5 (**A**; orange) and pH 7 (**B**; blue) at 20 °C by laccase *T. versicolor.* Data points represent $\overline{{x }_{\mathrm{r}}}$ ± *s*_r_. Each data point was determined at least in duplicate. APAP degradation after ozonation was analyzed in quadruplicate during the first 4 h

The kinetics of the enzymatic degradation of residual APAP in the ozonated matrix were analyzed and compared to the degradation kinetics of pure APAP solution. For the model wastewater, APAP was ozonated for 10 min to reach the maximum ecotoxicity. As depicted in Fig. 2A, APAP degradation at pH 5 by laccase was slightly slower in the ozonated solution than in the pure APAP solution. This was confirmed by the single exponential rate constants (Table S2) and by the analysis of multimer formation (Fig. S1). On the contrary, enzymatic APAP removal kinetics at pH 7 in the ozonated solution deviated more strongly from the reference (Fig. 2B). Within the first 4 h, no degradation of APAP (Fig. 2B) and no oligomer formation (Fig. S2B) took place, resembling a kinetic lag phase before the single exponential decay with a similar rate commenced (Table S2). The origin of the lag phase could only be explained after the enzymatic conversion of the ozonation by-products was resolved.

### Post-treatment of the ozonation transformation products by laccase

In the next step, the enzymatic degradation of the ozonation products TP 111 and TP 168 was analyzed. As phenolic compounds, the products fit into the substrate spectrum of laccases.

**Fig. 3:**
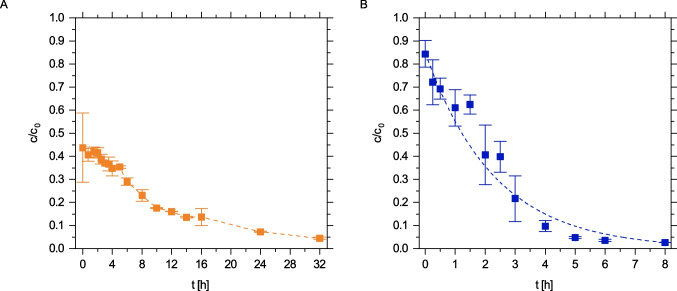
Degradation of the transformation product TP 168 at pH 5 (**A**; orange) and pH 7 (**B**; blue) at 20 °C by laccase of *T. versicolor.* Data points represent $\overline{{x }_{\mathrm{r}}}$ ± *s*_r_. Each data point was determined at least in quadruplicate during the first 4 h and at least in duplicate thereafter

The most abundant transformation product, TP 168, was successfully removed by laccase treatment (Fig. 3). However, in contrast to the activity optimum of the laccase, complete degradation was achieved earlier at pH 7 than at pH 5. At pH 5, 60% of TP 168 was removed rapidly within the first 20 min, but degradation was very slow thereafter, so that removing 95% of TP 168 required 32 h (Fig. 3A).

Interestingly, the lag phase of the respective APAP degradation kinetics coincided with the time required for 85% removal of TP 168 (4 h) at pH 7 (Fig. 2B). In order to investigate the interplay of TP 168 and APAP removal, degradation of the isolated reference compound 3-hydroxy-acetaminophen, i.e., TP 168, by laccase was analyzed.

The kinetics of TP 168 degradation in buffer and in the ozonation mixture were almost identical (Fig. S3). On the contrary, TP 168 strongly affected the degradation kinetics of other substrates, which was confirmed with a kinetic assay. Here, a lag phase of syringaldazine conversion was observed in the presence of TP 168, which increased in length with increasing TP 168 concentration (Fig. S4). This was a strong indication that TP 168 is a preferred substrate of laccase *T. versicolor* which was exclusively converted by the enzyme before any other substrate under investigation was accepted. Hence, the conversion of TP 168 was the reason for the lag phase in APAP depletion kinetics discussed above. This finding was rationalized by the calculated redox potentials, revealing a lower redox potential of TP 168 as compared to APAP at neutral conditions (Table S3). Besides the lag phase, no impact of TP 168 on the Michaelis–Menten parameters at pH 7 was found which confirmed the prior conversion of TP 168 (Table S4). The fact that the lag phase in APAP kinetics was not observed at pH 5 was justified by the fast depletion of TP 168.

**Fig. 4:**
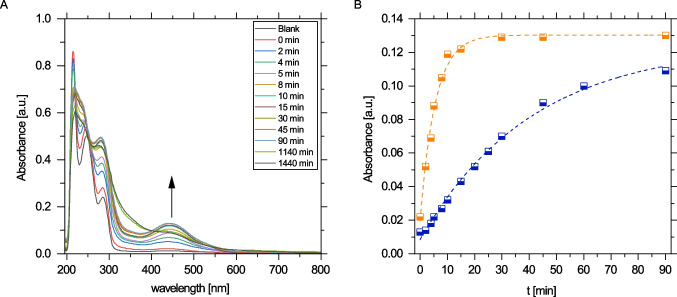
UV–Vis spectroscopic analysis of the degradation of pure TP 168 at 20 °C by laccase of *T. versicolor*: Time-dependent UV/Vis spectra during laccase treatment at pH 5 (**A**) and kinetic analysis of the formation of the product with an adsorption maximum of 450 nm at pH 5 (orange) and pH 7 (blue) (**B**)

The kinetics of the initial fast degradation of TP 168 at pH 5 were further analyzed by UV/Vis absorption spectroscopy, which provides a better time resolution than LC–MS. The absorption spectra changed significantly in the first 30 min (Fig. 4), thus confirming that fast conversion took place in the dead time of the LC–MS experiment. The UV/Vis spectra featured a new prominent band at approximately 450 nm upon enzymatic conversion of TP 168, which was attributed to the $n\to {\pi }^{*}$ transition of quinone species formed via oxidation of TP 168. Specifically, 4-acetamido-o-benzoquinone has been identified previously as the oxidation product of APAP by tyrosinase giving rise to similar UV/Vis spectra as obtained here (Fig. 4A) (Valero et al. 2003). The kinetic analysis yielded an approximately tenfold faster product formation at pH 5 than at pH 7 (Fig. 4B, Fig. S5, and Table S5).

A higher initial rate of enzymatic conversion of TP 168 at pH 5 than at neutral conditions could easily be explained by the pH optimum of the enzyme. However, the collapse in conversion after the initial fast reaction was surprising. A product inhibition was excluded by kinetic analysis using the syringaldazine activity assay (Table S4).

**Fig. 5:**
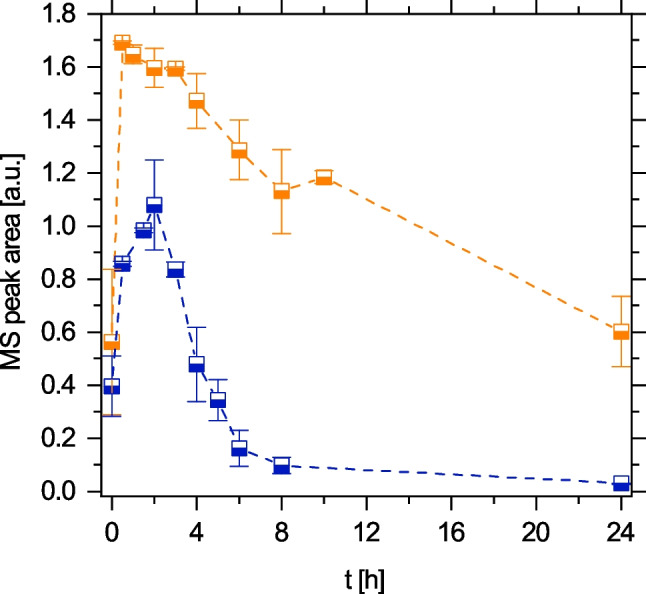
Formation of the secondary transformation product TP^2^ 166 ([M + H]^+^ 166.0518, RT 5.7 min) via the degradation of pure TP 168 by laccase *T. versicolor* at pH 5 (orange) and pH 7 (blue). Data points represent $\overline{{x }_{\mathrm{r}}}$ ± *s*_r_. Each data point was determined at least in triplicate

The hypothesis that 4-acetamido-o-benzoquinone was the product of laccase treatment of TP 168 was confirmed by HPLC-HRMS analysis, where a laccase product [M + H^+^] with an exact mass of 166.0518 was found (TP^2^ 166; Fig. 5). Interestingly, TP^2^ 166 accumulated to higher concentrations and during a more extended reaction period at pH 5 than at pH 7.

**Fig. 6:**
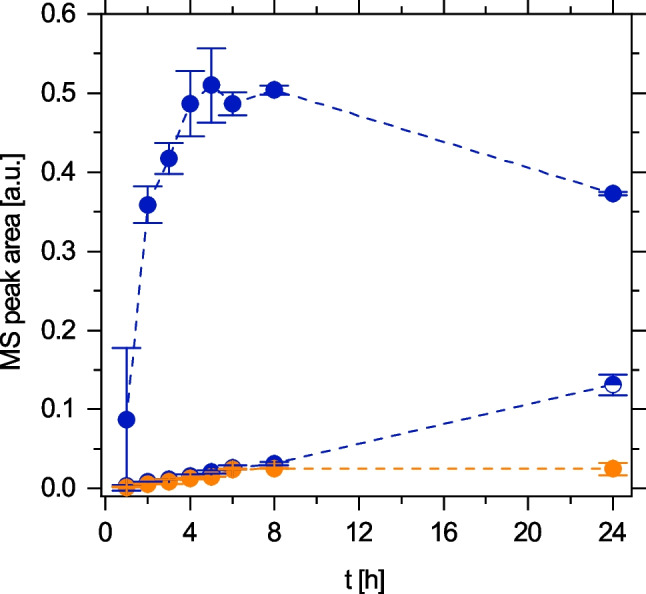
Formation of multimer products [M + H]^+^ 392.09 (half-filled circles) and [M + H]^+^ 449.10 (circles) during the laccase treatment of pure TP 168 at pH 5 (orange) and pH 7 (blue). Data points represent $\overline{{x }_{\mathrm{r}}}$ ± *s*_r_. Each data point was determined at least in duplicate

In order to reveal the different mechanisms of enzymatic conversion of TP 168 at the studied pH values, secondary transformation products generated by laccase treatment of TP 168 were determined by HPLC-HRMS. Suggestions for the molecular structure of the most abundant products TP^2^ 166, TP^2^ 392, and TP^2^ 449 are provided in Table S6. All detected products apart from TP^2^ 166 are oligomers. Figure 6 shows that oligomer formation and subsequent precipitation as insoluble polymers were primarily observed at pH 7, whereas oligomeric products were scarce at pH 5. This finding was bolstered by the comparison of the UV/Vis spectra at pH 5 (Fig. 4A) and pH 7 (Fig. S5), where additional scattering was superimposed on the band structure at pH 7, reflecting dispersed particles. We conclude that polymerization is favored at pH 7 whereas the quinoid product TP^2^ 166 dominates at pH 5.

It is important to note that the final products of laccase-catalyzed reactions result from the nature of the enzymatically formed radical and its subsequent chemical reaction of the radical to stable products. Typical reaction pathways are dimerization via C–C − or C–O–C bond, redox shuffle with non-substrates, and (de-)protonation (Kudanga et al. 2011).

Moreover, the formation of reactive oxygen species as side products of the substrate oxidation has been reported, including superoxide, hydrogen peroxide, hydroxyl radicals, and singlet oxygen, which can undergo further reactions with the organic substrates (Huang and Yang 2022; Ulas et al. 2016).

Therefore, chemical processes need to be considered to determine the root cause of the different products and kinetics observed in response to the different pH values in the enzymatic treatment. Few data on oxidation pathways of hydroxy-acetaminophen are available, but the chemical oxidation processes of catechol and dopamine have been intensely studied (Slikboer et al. 2015; Salomäki et al. 2018). The oxidation of catechol derivatives involves the two redox states semiquinone and quinone. The semiquinone can (1) oligomerize via C–C (or C–O) coupling or (2) disproportionate to quinone and hydroquinone (Pezzella et al. 2013). If the disproportionation predominates, a defined part of the catechol is immediately oxidized to o-quinone to reach thermodynamic equilibrium (Salomäki et al. 2018). For further conversion of the hydroquinone, the quinone has to be removed from the thermodynamic equilibrium. In the metabolic degradation of acetaminophen, quinones are depleted via Michael addition with glutathione (Mazaleuskaya et al. 2015).

In the absence of nucleophiles, o-quinones are nearly dead-end products (Tentscher et al. 2018) as autooxidation is very slow. The removal via the reverse reaction, i.e., comproportionating to semiquinone and potential coupling reactions thereof, is blocked by the presence of the laccase, which continuously reoxidizes all species to the quinone. The latter hypothesis was supported by experiments with benzoquinone, which was more stable towards auto-degradation in the presence of laccase (Fig. S9).

**Fig. 7:**
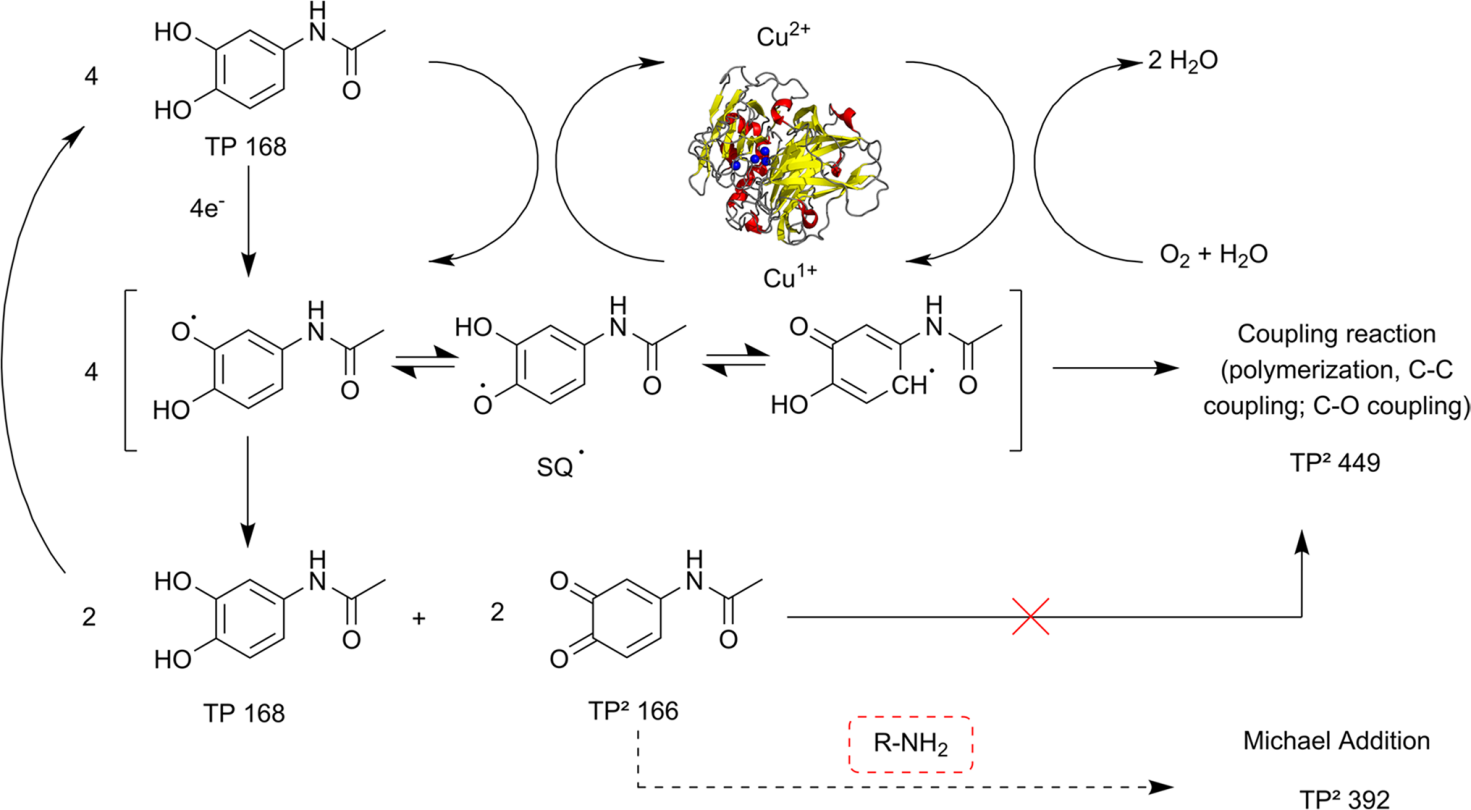
Schematic representation of the suggested mechanism of laccase-catalyzed degradation of TP 168. First, the semiquinone radical of TP 168 (SQ^•^) is formed which can either form oligomers or disproportionate to TP 168 and the quinone TP^2^ 166

The respective mechanism transferred to the enzymatic degradation of TP 168 is illustrated in Fig. 7. The formation of oligomers predominantly occurs if the semiquinone radical is stable against disproportionation. Semiquinone stability increases with increasing pH (Song and Buettner 2010). Consequently, the initial rapid kinetic phase of TP 168 degradation at pH 5 is attributed to the formation of the quinone by disproportionation. Thus, the fast conversion of hydroquinone ceases at its equilibrium value. We suggest that, in contrast, at pH 7 the stability of the semiquinone is sufficient to allow its oligomerization. The slow intermediate kinetic phase in the degradation of TP 168 (Fig. 3A) is interpreted by the slow removal of the quinone via autooxidation and Michael addition of the free amine generated by hydrolysis.

Once a number of oxygenated species have been formed, the removal of the quinone as well as TP 168 is accelerated due to potential coupling partners leading to the third kinetic phase of TP 168 removal.

The contribution of reactive hydrogen species, especially after the conversion of good substrates after 4 h, is likely, but cannot be proven.

These findings could be extended to the enzymatic degradation of the ozonation product TP 111 which was also removed more efficiently at pH 7 than at pH 5. At pH 7, a removal efficiency of (78 ± 4)% (Fig. S10) was achieved after 48 h. In accordance with the analysis of TP 168 degradation, TP 111 removal of 25% at pH 5 is assigned to the quinone pathway of the 1,4- and 1,2-isomer of TP 111 (Sun et al. 2013), whereas the higher removal efficiency at pH 7 is suggested to result from the oligomer pathway.

**Fig. 8:**
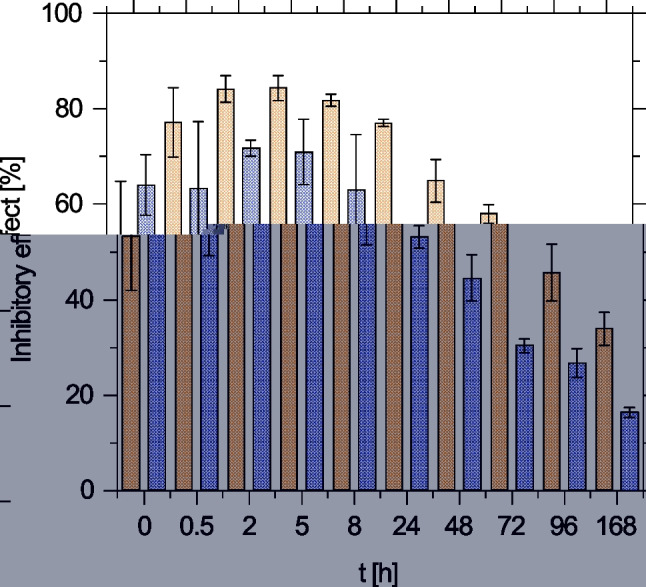
Development of acute toxicity during treatment of ozonated APAP solution at pH 5 (orange bars) and pH 7 (blue bars) with laccase *T. versicolor* after 5-min contact time with *A. fischeri*. Data represent $\overline{x }$ ± SD. Each data point was determined in quadruplicate

The ecotoxicity assay using* A. fischeri* (Fig. 8) revealed that laccase treatment reduced the ecotoxicity of ozonated APAP. However, the ecotoxicity increased at the beginning of the laccase treatment, which was more pronounced and persistent at pH 5 than at pH 7. A significant reduction of the initial toxicity of the ozonated solution by laccase treatment was observed after 24 at pH 7 (Table S11), whereas this was only achieved after 168-h laccase treatment at pH 5 (Table S12). Here, ecotoxicity is predominantly attributed to the presence of the quinone TP^2^ 166 (Fig. 6; Table S13). This emphasizes that neutral conditions are recommended for the laccase treatment of the ozonation products to harmless products despite the enzyme activity maximum at acidic conditions.

## Conclusion

A new method of post-treatment of ozonated wastewater for the removal of dangerous ozonation products by the application of enzyme catalysis is presented and successfully applied to the remediation of acetaminophen model wastewater. The approach takes advantage of the fact that phenolic ozonation products have the highest ecotoxicological potential and are particularly susceptible to enzymatic elimination by laccase from *T. versicolor*. Thus, a significant reduction in the ecotoxicity of an ozonated APAP solution was achieved through enzymatic post-treatment. However, optimum remediation is not reached in the pH activity optimum of the enzyme, because the (redox) chemistry following laccase-induced radical formation is also influenced by the pH. In the case of acetaminophen, the fast reaction to dead-end quinone products needs to be suppressed for oligomerization to occur. This can be realized by performing the laccase treatment under neutral conditions, where the enzymatic treatment safely converts the harmful ozonation products to insoluble and harmless oligomers.

## Supplementary information

Below is the link to the electronic supplementary material.

Supplementary file1 (PDF 1.83 MB)

## Data Availability

The data supporting the findings of this study are available within the article and its supplementary materials.
