# Supplementary material for: Enzymatic post-treatment of ozonation: laccase-mediated removal of the by-products of acetaminophen ozonation
Source: Environ Sci Pollut Res Int. 2023 Feb 28;30(18):53128–39. doi: 10.1007/s11356-023-25913-w (PMC10119220; doi:10.1007/s11356-023-25913-w)
Supplement: Supplementary file 1 — Supplementary file1 (PDF 1.83 MB) [file 11356_2023_25913_MOESM1_ESM.pdf]

Supplementary information

Enzymatic post-treatment of ozonation: laccase-mediated removal of the by-products of acetaminophen ozonation

*Dorothee Schmiemann[1,2], Lisa Hohenschon[1,3], Indra Bartels[1,4], Andrea Hermsen[1,5], Felix Bachmann[6], Arno Cordes[6], Martin Jäger[1], Jochen Stefan Gutmann[2,7] and Kerstin Hoffmann-Jacobsen[1]**

[1] Department of Chemistry and Institute for Coatings and Surface Chemistry, Niederrhein University of Applied Sciences, Adlerstr. 32, 47798 Krefeld, Germany

[2] Institute of Physical Chemistry and CENIDE (Center for Nanointegration), University Duisburg-Essen, Universitätsstraße 5, 45141 Essen, Germany

[3] Present Address: wfk-Cleaning Technology-Institute e.V., Campus Fichtenhain 11, 47807 Krefeld, Germany

[4] Faculty of Chemistry, Instrumental Analytical Chemistry, University of Duisburg-Essen, Universitätsstraße 5, 45141 Essen, Germany

[5] Present Address: Institute of Theoretical Chemistry, University Duisburg-Essen, Universitätsstraße 5, 45141 Essen, Germany

[6] ASA Spezialenzyme GmbH, Am Exer 19c, 38302 Wolfenbüttel, Germany

[7] Deutsches Textilforschungszentrum Nord-West gGmbH, Adlerstr. 1, 47798 Krefeld, Germany

* Kerstin.Hoffmann-Jacobsen@hs-niederrhein.de

1.1 Correlation analysis of the acute toxicity during ozonation

The Pearson correlation coefficient (*r*) of the relative peak area of the ozonation products and the inhibitory effect of the solution on *A. fischeri* luminescence after the indicated incubation time was calculated. As depicted in Tab. S1, a correlation of the inhibitory effect with the MS peak area of TP 168 was found at all incubation times.

Tab. S1 Pearson correlation coefficient of the relative MS peak area of the indicated transformation products and the inhibitory effect on the bioluminescence of *A. fischeri* after 5, 15 and 30 min incubation and the corresponding p values. **Correlations with a significance level of 0.05** are depicted in bold numbers.

	r_5	p_5	r_{15}	p_{15}	r_{30}	p_{30}
TP 168	**0.89**	0.045	**0.99**	0.0012	**0.98**	0.0034
TP 111	0.18	0.77	0.28	0.64	0.41	0.49
TP 200	0.22	0.72	0.13	0.84	0.002	1.00

1.2 Degradation of APAP by laccase *T. versicolor*

Tab. S2 Rate constants of APAP degradation by laccase from *T. versicolor* at pH 5 and 7 with and without previous ozone treatment.

	pure solution		after ozonation	
pH	5	7	5	7
k [h^{-1}]	1.04 ± 0.008	0.15 ± 0.003	0.78 ± 0.018	0.11 ± 0.001[a]
R^2	0.99	1.00	0.99	0.98

[a] exponential fit after 4 h lag phase

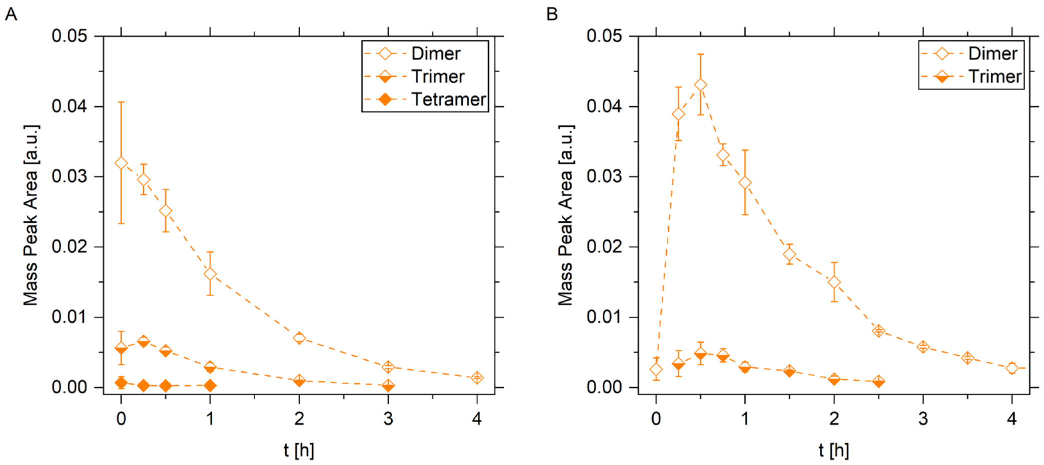

Fig. S1 Formation of APAP dimer ([M+H]$^+$ 301.10), trimer ([M+H]$^+$ 450.15) and tetramer ([M+H]$^+$ 599.40) without prior ozonation (A) and with prior ozonation (B) at pH 5 through the degradation of APAP at 20°C by laccase *T. versicolor*. Data points represent $\overline{x_r}$ ± s_r. Each data point was measured in triplicate.

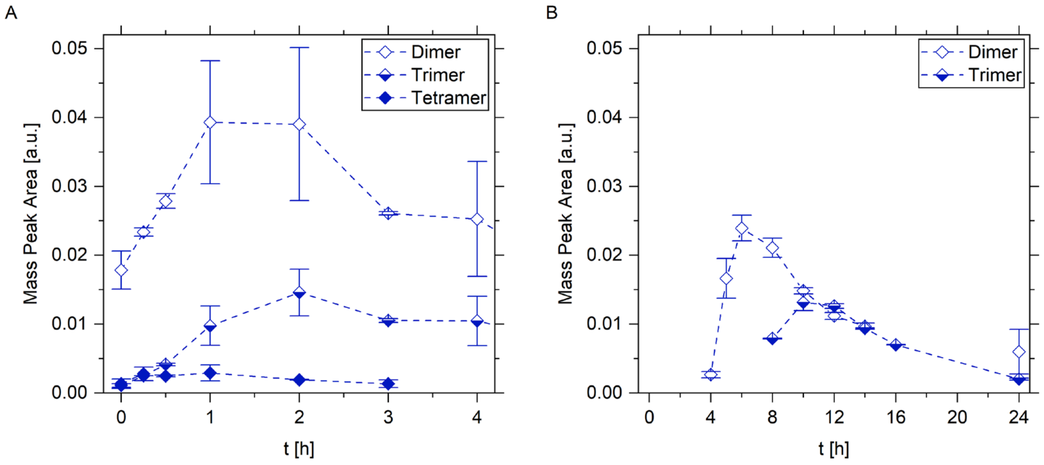

Fig. S2 Formation of APAP dimer ([M+H]$^+$ 301.10), trimer ([M+H] $^+$ 450.15) and tetramer ([M+H]$^+$ 599.40) without prior ozonation (A) and with prior ozonation (B) at pH 7 through the degradation of APAP at 20°C by laccase *T. versicolor*. Data points represent $\overline{x_r}$ ± s_r. Each data point was measured at least in duplicate.

1.3 Post-treatment of TP 168 by laccase *T. versicolor*

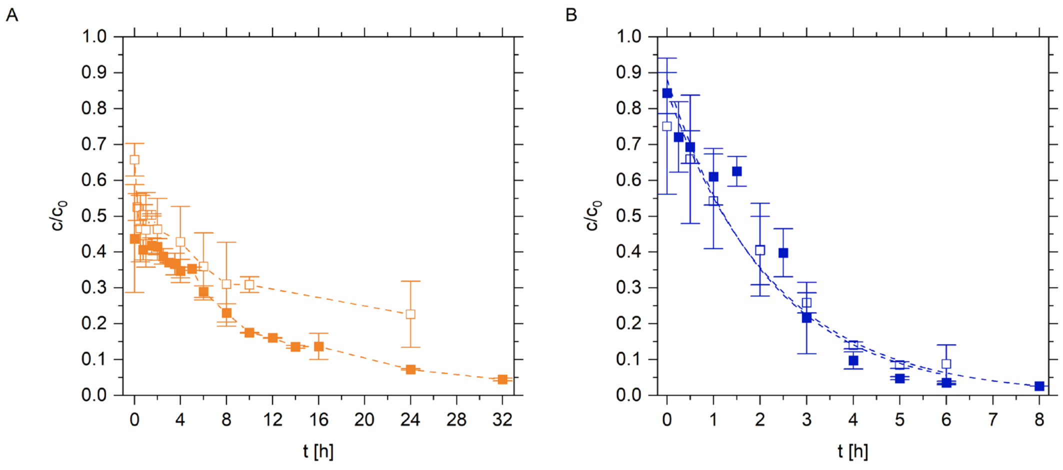

Fig. S3 Degradation of pure TP 168 without ozonation (filled squares) and with previous ozone treatment (unfilled squares) at pH 5 (A, orange) and pH 7 (B, blue) at 20°C by laccase *T. versicolor*. Data points represent $\overline{x_r} \pm s_r$. Each data point of TP 168 degradation without ozonation was measured at least in triplicate and TP 168 degradation with ozonation was measured at least in quadruplicate in the first 4 h.

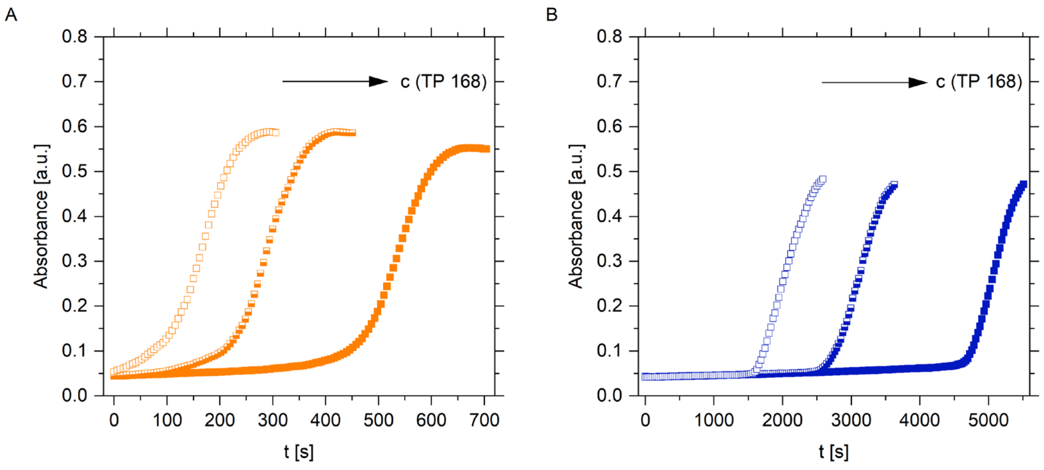

Fig. S4 Time traces of syringaldazine (c_0=10 µM) oxidation by laccase *T. versicolor* at different concentrations of TP 168 (55 µM filled squares; 27.80 µM half-filled squares and 13.90 µM unfilled squares) at pH 5 (A, orange) and pH 7 (B, blue); 30°C, plate reader.

1.4 Calculation of the redox potentials of the transformation products

For the computational calculations of the redox potentials, gaussian 16 program package was used (Frisch et al. 2016). Complete geometry optimization was performed using density functional theory with the functional B3LYP and the basis set 6-311+(2d, p) (Becke 1993, Stephens et al. 1994). Based on a frequency calculation and the absence of imaginary frequencies, the optimized geometries are proven to be in local minima of the potential energy surface.

For the calculation of the redox potentials, calculations were also carried out in an aqueous solution. The solvent effect was modelled by SCRF-PCM method (Miertuš et al. 1981). The Gibbs Energies were corrected with the zero-point energies and the redox potential was calculated with Born-Haber-cycle and calculated against NHE (2019).

Tab. S3 Calculated redox potentials (B3LYP/6-311+(2d, p), PCM) of APAP, TP 168 and TP 111 versus the potential of the normal hydrogen electrode (NHE).

**substrate**	**E^0 (V vs. NHE)**
APAP	1.20
TP 168	1.11
TP 111_{meta}	1.60
TP 111_{ortho}	1.53
$TP111_{para}$	1.31

1.5 Michaelis Menten kinetics of laccase *T. versicolor*

In Michaelis Menten kinetics, the dependency of the initial rate of catalysis on the substrate concentration [S] is described by Eq. S1:

$$v = v_{max} \cdot \frac{[S]}{K_m + [S]} \qquad \text{(Eq. S1)}$$

with v_{max} being the maximum rate and K_m the Michaelis Menten constant. The turnover number, k_{cat}, is calculated by equation S2:

$$k_{cat} = \frac{v_{max}}{[E]} \qquad \text{(Eq. S2)}$$

with $[E]$ being the enzyme concentration.

Tab. S4 Results of the Michaelis Menten analysis: Maximum rate (v_{max}), Michaelis Menten constant (K_m) and the turnover number (k_{cat}) of laccase *T. versicolor* at pH 5 and pH 7, substrate: syringaldazine. The goodness of the fit which has been performed with the software "OriginPro® 2020 SR1" is quantified by the reduced standard error of the regression (χ^2 reduced). The activity of each substrate concentration was determined in triplicate.

**buffer**	**pH 5**	**pH 7**
v_{max} [mol/L*s]	$4.31\cdot10^{-7} \pm 6.17\cdot10^{-8}$	$1.90\cdot10^{-8} \pm 4.82\cdot10^{-10}$
K_m [mol/L]	$2.08\cdot10^{-5} \pm 7.02\cdot10^{-6}$	$2.02\cdot10^{-6} \pm 6.57\cdot10^{-7}$
k_{cat} [1/s]	42.3 ± 6.06	1.86 ± 0.05
χ^2 reduced	$2.08\cdot10^{-11}$	$3.39\cdot10^{-11}$
**ozonated APAP**	**pH 5**	**pH 7**
v_{max} [$mol\cdot L^{-1}\cdot s^{-1}$]	$5.58\cdot10^{-7} \pm 1.38\cdot10^{-7}$	$2.87\cdot10^{-8} \pm 1.93\cdot10^{-9}$
K_m [$mol\cdot L^{-1}$]	$4.52\cdot10^{-5} \pm 1.86\cdot10^{-5}$	$5.47\cdot10^{-6} \pm 1.93\cdot10^{-6}$
k_{cat} [s^{-1}]	54.77 ± 13.60	2.82 ± 0.19
χ^2 reduced	$1.18\cdot10^{-11}$	$1.63\cdot10^{-11}$
**30 µmoL TP 168**	**pH 5**	**pH 7**
v_{max} [$mol\cdot L^{-1}\cdot s^{-1}$]	$5.57\cdot10^{-7} \pm 1.33\cdot10^{-7}$	$2.38\cdot10^{-8} \pm 9.85\cdot10^{-10}$
K_m [$mol\cdot L^{-1}$]	$3.82\cdot10^{-5} \pm 1.59\cdot10^{-5}$	$3.70\cdot10^{-6} \pm 1.16\cdot10^{-6}$
k_{cat} [s^{-1}]	54.7 ± 13.02	2.34 ± 0.10
χ^2 reduced	$1.69\cdot10^{-11}$	$2.21\cdot10^{-11}$
**60 µmoL TP 168**	**pH 5**	**pH 7**
v_{max} [$mol\cdot L^{-1}\cdot s^{-1}$]	$5.07\cdot10^{-7} \pm 6.84\cdot10^{-8}$	$2.09\cdot10^{-8} \pm 2.36\cdot10^{-10}$
K_m [$mol\cdot L^{-1}$]	$3.42\cdot10^{-5} \pm 8.68\cdot10^{-6}$	$4.68\cdot10^{-6} \pm 3.54\cdot10^{-7}$
k_{cat} [s^{-1}]	49.8 ± 6.72	2.06 ± 0.02
χ^2 reduced	$6.26\cdot10^{-12}$	$2.07\cdot10^{-13}$

The slight increase in K_m indicates minor matrix effects of the ozonated mixture on the affinity of syringaldazine to the laccase. As shown in Fig. S3, these minor matrix effects only marginally inflict the degradation kinetics of the preferred substrate TP 168.

1.6 UV/Vis-spectroscopic analysis of TP 168 transformation at pH 7

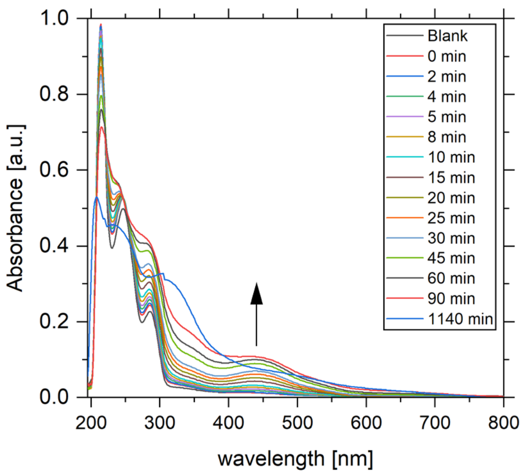

Fig. S5 UV-VIS spectroscopic analysis of the degradation of pure TP 168 (10 mg/L) at 20°C by laccase *T. versicolor*: Time-dependent UV/Vis spectra during laccase treatment at pH 7.

Tab. S5 Kinetic constants of the formation of the product with an absorption maximum 450 nm during the degradation of pure TP 168 at pH 5 and pH 7.

	pH 5	pH 7
k [min^{-1}]	0.18 ± 0.0144	0.02 ± 0.0021
R^2	0.99	0.99

1.7 Transformation products of TP 168

The transformation products formed by laccase treatment of TP 168 were analysed by MS^2 analysis with a collision energy of 30 eV. The spectra are shown in Fig. S6-S8 and the mass of the $[M+H]^+$ signals and of the fragments are given in Tab. S6. Based on the exact mass and the isotope pattern of the $[M+H]^+$ signal, a molecular mass was deduced.

The molecular structure is in line with the fragmentation pattern found. Due to the lack of ring-opening fragments the substitution pattern has not been resolved.

The fragmentation pattern showed some similarities with the MS^2 analysis of APAP dimer formation. Following a previous study (Lu et al. 2009), the elimination of acetic acid (m/z = 60) was assigned to the presence of acetamide groups and used to determine the number thereof.

Tab. S6 Exact and accurate masses of the $[M+H]^+$ ions and chemical formula of the products of TP 168 oxidation by laccase *T. versicolor*. The structure of TP^2 166 is unambiguously determined. The other structures are in line with all fragments found (MS^2).

**compound**	**$[M+H]^+$ (exact)**	**$[M+H]^+$ (accurate)**	**MS^2**	**Proposed structure**
TP^2 166	166.0498	166.0526	124.04	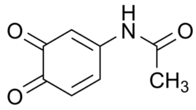
			108.04	
			96.04	$C_8H_7NO_3$
			78.03	
			68.05	
			51.02	
			43.02	
TP^2 392	392.0877	392.0842	374.07	
			350.08	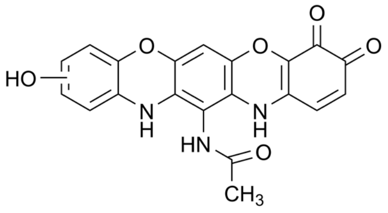
			332.06	
			318.08	
			290.08	
			276.07	$C_{20}H_{13}N_3O_6$
			248.07	

TP[2] 449	449.0979	449.1058	407.09<br>389.08<br>365.08<br>347.07<br>244.06<br>217.05<br>191.04<br>147.05	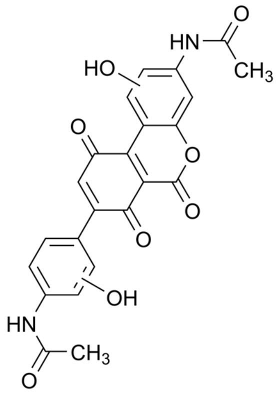<br>$C_{23}H_{16}N_2O_8$

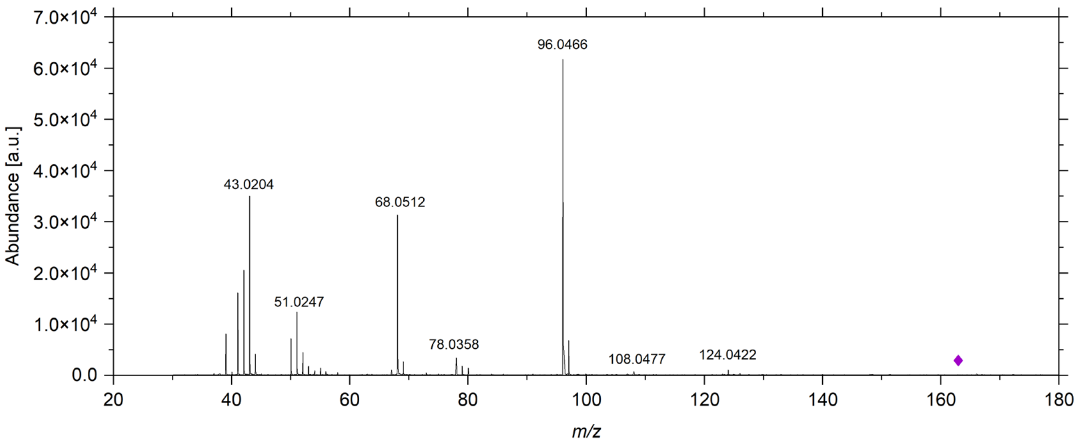

Fig. S6 MS^2 spectrum of $[M+H]^+$ 166.0526, collision energy 30 eV.

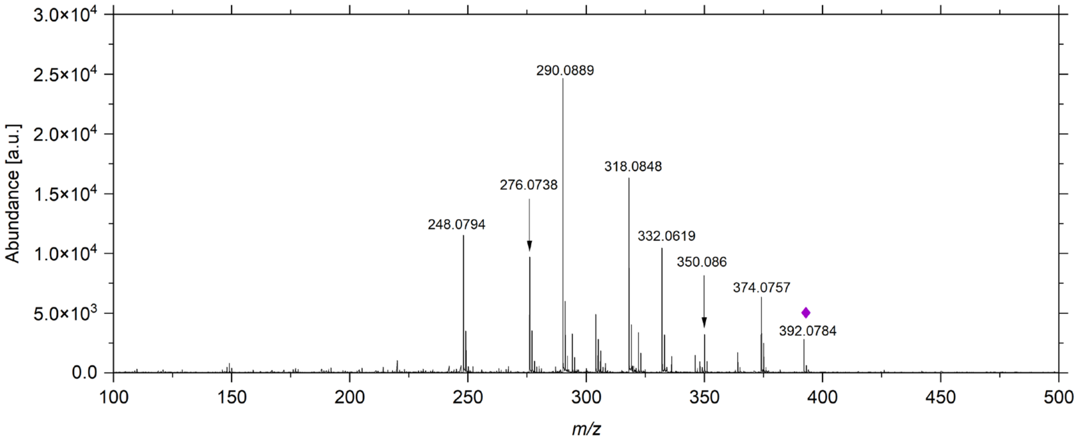

Fig. S7 MS^2 spectrum of $[M+H]^+$ 392.0784, collision energy 30 eV.

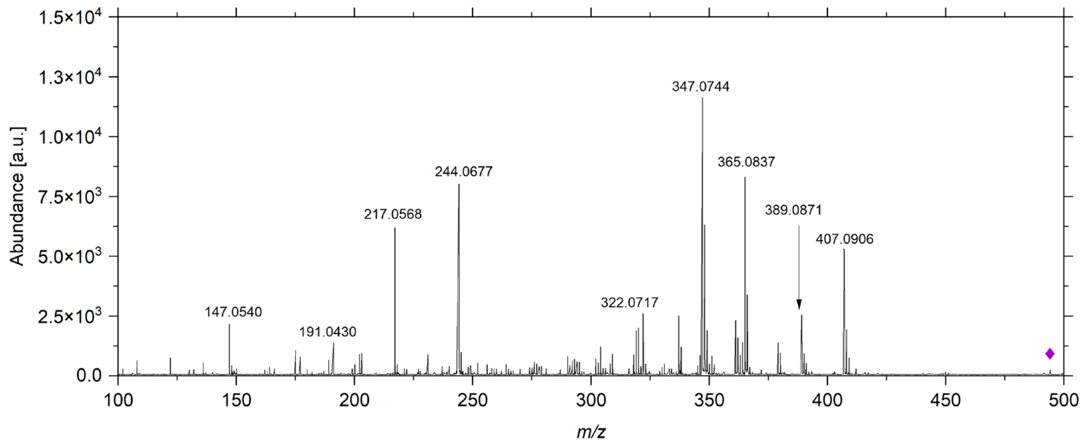

Fig. S8 MS^2 spectrum of $[M+H]^+$ 449.1100, collision energy 30 eV.

1.8 Reverse reaction by laccase *T. versicolor*

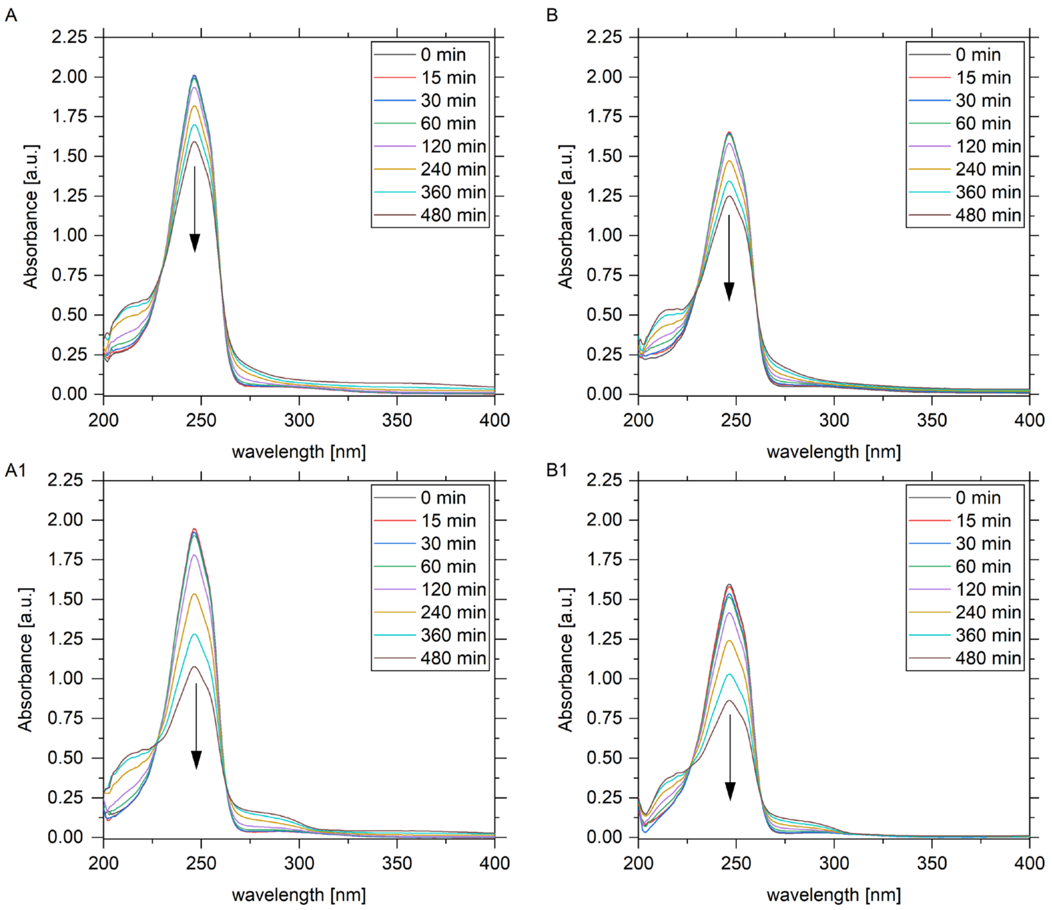

Fig. S9 UV-VIS spectra of benzoquinone (10 mg/L) in the presence (A/B) and absence (A1/B1) of laccase *T. versicolor* at pH 5 (left) and pH 7 (right), 20 °C.

1.9 Post-treatment of TP 111 by laccase *T. versicolor*

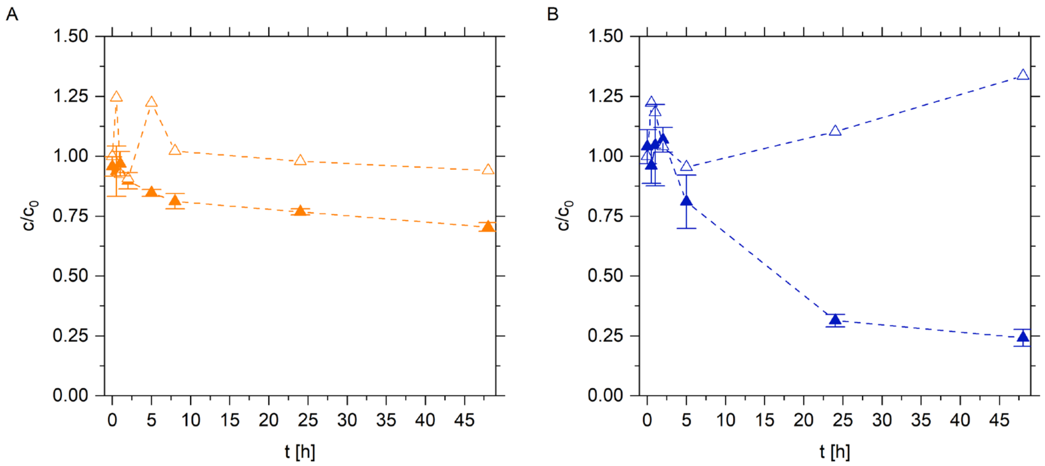

Fig. S10 Degradation of TP 111 after ozonation at pH 5 (A, orange) and pH 7 (B, blue) at 20°C by laccase from *T. versicolor*. Unfilled triangles depict the reference in the absence of laccase. The reference shows that TP 111 is formed at the beginning of the experiment by autooxidation. This explains the slow onset of the degradation by laccase treatment. Data points represent $\overline{x_r} \pm s_r$. Each data point was measured at least in duplicate.

1.10 Statistical analysis of the toxicity reduction by laccase treatment

The ecotoxicity data during laccase treatment of the ozonated solution was subjected to statistical analysis.

1.10.1 Two-sample F-test

Variance homogeneity was tested by comparing the F-test statistics with the critical F- value of a significance level of 0.05. F-test statistics were calculated with Eq. S3:

$$F = \frac{s_1^2}{s_2^2} \qquad \text{(Eq. S3)}$$

with s^2 depicting the variances. If F is smaller than the critical value F_{crit}, the variances differ only by coincidence.

Tab. S7 F-test of variance homogeneity of the toxicity at pH 5 and pH 7 at the given time point of the laccase treatment of ozonated APAP (s. Fig. 8). Bold numbers indicate a **significant difference of variances**.

t [h]	F	F_{crit}
0	3.19	9.28
0.5	3.72	9.28
2	2.57	9.28
5	6.41	9.28
**8**	**75.98**	**9.28**
24	8.21	9.28
48	1.14	9.28
72	1.90	9.28
96	3.91	9.28
**168**	**10.89**	**9.28**

Tab. S8 F-test of variance homogeneity in the time series of laccase treatment of ozonated APAP at pH 5 with respect to the initial toxicity of the ozonated solution (s. Fig. 8). Bold numbers indicate a **significant difference of variances**.

t [h]	F	F_{crit}
0	-	-
0.5	2.44	9.28
**2**	**16.89**	**9.28**
5	18.12	9.28
**8**	**74.48**	**9.28**
**24**	**194.78**	**9.28**
48	6.51	9.28
**72**	**33.41**	**9.28**
**96**	**3.73**	**9.28**
168	11.05	9.28

Tab. S9 F-Test of variance homogeneity in the time series of laccase treatment of ozonated APAP at pH 7 with respect to the initial toxicity of the ozonated solution (s. Fig. 8). Bold numbers indicate a **significant difference of variances**.

t [h]	F	F_{crit}
0	-	-
0.5	4.85	9.28
**2**	**13.61**	**9.28**
5	1.13	9.28
8	3.25	9.28
24	7.45	9.28
48	1.79	9.28
**72**	**19.86**	**9.28**
96	4.58	9.28
**168**	**37.74**	**9.28**

1.10.2 t-test

The development of acute toxicity during the treatment of ozonated APAP solution with laccase was investigated with respect to two hypotheses: (1) The toxicity during the treatment of ozonated APAP solution with laccase *T. versicolor* is higher at pH 5 than at pH 7, (2) the acute toxicity decreased by the treatment of ozonated APAP solution with laccase. The hypotheses were examined with the two-tailed t-test (variances homogeneity) and the two-tailed t-test according to Welch (variances inhomogeneity).

1.10.2.1 Two-tailed t-test

The t statistics of the experiment was calculated according to Eq. S4:

$$t = \frac{|\bar{x}_1 - \bar{x}_2|}{s_d} \cdot \sqrt{\frac{n_1 \cdot n_2}{n_1 + n_2}} \qquad \text{(Eq. S4)}$$

with the mean values $\bar{x}_1$ and $\bar{x}_2$, and the number of measurements n_1, n_2. The weighted average standard deviation s_d was calculated via Eq. S5 from the standard deviations s_1, s_2.

$$s_d = \sqrt{\frac{(n_1 - 1) \cdot s_1^2 + (n_2 - 1) \cdot s_2^2}{n_1 + n_2 - 2}} \qquad \text{(Eq. S5)}$$

1.10.2.2 Two-tailed t-test according to Welch

The experimental t-value was calculated according to Eq. 6:

$$t = \frac{|\bar{x}_1 - \bar{x}_2|}{\sqrt{\frac{s_1^2}{n_1} + \frac{s_2^2}{n_2}}} \qquad \text{(Eq. S6)}$$

The number of degrees of freedom (f) was calculated with Eq. S7:

$$f = \frac{\left(\frac{s_1^2}{n_1} + \frac{s_2^2}{n_2}\right)^2}{\frac{\left(\frac{s_1^2}{n_1}\right)^2}{n_1 - 1} + \frac{\left(\frac{s_2^2}{n_1}\right)^2}{n_2 - 1}} \qquad \text{(Eq. S7)}$$

t-values were compared with the critical t value of a significance level of 0.05. For comparison all data points were analysed by both tests, the two-tailed t-test and the extended t-test according to Welch.

Tab. S10 t-test of toxicity values in the time series of laccase treatment of ozonated APAP at pH 5 and pH 7 (s. Fig. 8). Bold numbers indicate that a **significant difference of the mean values is found**. Data lacking variance homogeneity are shown in italics.

	two-tailed t-test			t-test according to Welch		
t [h]	t	t_{crit}	f	t	t_{crit}	f
0	1.63	2.45	6	1.63	2.57	5
0.5	1.74	2.45	6	1.74	2.57	5
**2**	**7.55**	**2.45**	**6**	**7.55**	**2.57**	**5**
**5**	**3.67**	**2.45**	**6**	**3.67**	**2.78**	**4**
8	***3.24***	***2.45***	***6***	***3.24***	***3.18***	***3***
**24**	**19.25**	**2.45**	**6**	**19.25**	**2.78**	**4**
**48**	**6.23**	**2.45**	**6**	**6.23**	**2.45**	**6**
**72**	**22.54**	**2.45**	**6**	**22.54**	**2.57**	**5**
**96**	**5.69**	**2.45**	**6**	**5.69**	**2.78**	**4**
168	***9.74***	***2.45***	***6***	***9.74***	***2.78***	***4***

Tab. S11 t-test of the toxicity values at different reaction times of laccase treatment of ozonated APAP at pH 7 with respect to the initial toxicity of ozonated APAP at 0 h (s. Fig. 8). Bold numbers indicate that a **significant reduction of the toxicity** with respect to the initial ozonated solution is found. Data lacking variance homogeneity are shown in italics.

	two-tailed t-test			t-test according to Welch		
t [h]	t	t_{crit}	f	t	t_{crit}	f
0	-	-	-	-	-	-
0.5	0.10	2.45	6	0.10	2.78	4
2	*2.33*	*2.45*	*6*	*2.33*	*3.18*	*3*
5	1.48	2.45	6	1.48	2.45	6
8	0.16	2.45	6	0.16	2.57	5
**24**	**3.20**	**2.45**	**6**	**3.20**	**2.78**	**4**
**48**	**4.88**	**2.45**	**6**	**4.88**	**2.45**	**6**
72	***10.25***	***2.45***	***6***	***10.25***	***3.18***	***3***
**96**	**10.55**	**2.45**	**6**	**10.55**	**2.78**	**4**
168	***14.67***	***2.45***	***6***	***14.67***	***3.18***	***3***

Tab. S12 t-test of the toxicity values at different reaction times of laccase treatment of ozonated APAP at pH 5 with respect to the initial toxicity of ozonated APAP at 0 h (s. Fig. 8). Bold numbers indicate that a **significant reduction of the toxicity** with respect to the initial ozonated solution is found. Data lacking variance homogeneity are shown in italics.

	two-tailed t-test			t-test according to Welch		
t [h]	t	t_{crit}	f	t	t_{crit}	f
0	-	-	-	-	-	-
0.5	3.50	2.45	6	3.50	2.57	5
2	*5.23*	*2.45*	*6*	*5.23*	*3.18*	*3*
5	5.28	2.45	6	5.28	3.18	3
8	*4.94*	*2.45*	*6*	*4.94*	*3.18*	*3*
24	*4.13*	*2.45*	*6*	*4.13*	*3.18*	*3*
48	1.89	2.45	6	1.89	2.78	4
72	*0.79*	*2.45*	*6*	*0.79*	*3.18*	*3*
96	*1.20*	*2.45*	*6*	*1.20*	*2.57*	*5*
**168**	**3.26**	**2.45**	**6**	**3.26**	**2.78**	**4**

1.10.3 Correlation analysis of the acute toxicity during laccase treatment

The Pearson correlation coefficient (*r*) of the relative peak area of TP^2 166 and the inhibitory effect of the ozonated solution during laccase treatment on *A. fischeri* luminescence after the indicated incubation time was calculated. As depicted in Tab. S13, a moderate correlation was found with a significance level of ca. 0.15.

Tab. S13 Pearson correlation coefficient of the MS peak area of the transformation product TP^2 166 and the inhibitory effect on the bioluminescence of *A. fischeri* after 5, 15 and 30 min incubation and the corresponding p values.

	r_5	p_5	r_{15}	p_{15}	r_{30}	p_{30}
pH 5	0.67	0.14	0.67	0.15	0.66	0.16
pH 7	0.70	0.12	0.74	0.09	0.68	0.14

References

Becke AD (1993) Density-functional thermochemistry. III. The role of exact exchange. The Journal of Chemical Physics 98(7):5648–5652. doi: 10.1063/1.464913

Frisch MJ, Trucks GW, Schlegel HB, Scuseria GE, Robb MA, Cheeseman JR, Scalmani G, Barone V, Petersson GA, Nakatsuji H, Li X, Caricato M, Marenich AV, Bloino J, Janesko BG, Gomperts R, Mennucci B, Hratchian HP, Ortiz JV, Izmaylov AF, Sonnenberg JL, Williams, Ding F, Lipparini F, Egidi F, Goings J, Peng B, Petrone A, Henderson T, Ranasinghe D, Zakrzewski VG, Gao J, Rega N, Zheng G, Liang W, Hada M, Ehara M, Toyota K, Fukuda R, Hasegawa J, Ishida M, Nakajima T, Honda Y, Kitao O, Nakai H, Vreven T, Throssell K, Montgomery Jr. JA, Peralta JE, Ogliaro F, Bearpark MJ, Heyd JJ, Brothers EN, Kudin KN, Staroverov VN, Keith TA, Kobayashi R, Normand J, Raghavachari K, Rendell AP, Burant JC, Iyengar SS, Tomasi J, Cossi M, Millam JM, Klene M, Adamo C, Cammi R, Ochterski JW, Martin RL, Morokuma K, Farkas O, Foresman JB, Fox DJ (2016) Gaussian 16 Rev. C.01, Wallingford, CT

Lu J, Huang Q, Mao L (2009) Removal of acetaminophen using enzyme-mediated oxidative coupling processes: I. Reaction rates and pathways. Environmental science & technology 43(18):7062–7067. doi: 10.1021/es9002422

Miertuš S, Scrocco E, Tomasi J (1981) Electrostatic interaction of a solute with a continuum. A direct utilizaion of AB initio molecular potentials for the prevision of solvent effects. Chemical Physics 55(1):117–129. doi: 10.1016/0301-0104(81)85090-2

(2019) standard hydrogen electrode. In: Gold V (ed) The IUPAC Compendium of Chemical Terminology. International Union of Pure and Applied Chemistry (IUPAC), Research Triangle Park, NC

Stephens PJ, Devlin FJ, Chabalowski CF, Frisch MJ (1994) Ab initio calculation of vibrational absorption and circular dichroism spectra using density functional force fields. The Journal of physical chemistry 98(45):11623–11627
